# Control of mRNA stability contributes to low levels of nuclear poly(A) binding protein 1 (PABPN1) in skeletal muscle

**DOI:** 10.1186/2044-5040-3-23

**Published:** 2013-10-01

**Authors:** Luciano H Apponi, Anita H Corbett, Grace K Pavlath

**Affiliations:** 1 Department of Pharmacology, Emory University School of Medicine, Atlanta, GA, USA; 2 Department of Biochemistry, Emory University School of Medicine, Atlanta, GA, USA

**Keywords:** PABPN1, OPMD, Skeletal muscle, Muscular dystrophy, Polyalanine expansion

## Abstract

**Background:**

The nuclear poly(A) binding protein 1 (PABPN1) is a ubiquitously expressed protein that plays critical roles at multiple steps in post-transcriptional regulation of gene expression. Short expansions of the polyalanine tract in the N-terminus of PABPN1 lead to oculopharyngeal muscular dystrophy (OPMD), which is an adult onset disease characterized by eyelid drooping, difficulty in swallowing, and weakness in the proximal limb muscles. Why alanine-expanded PABPN1 leads to muscle-specific pathology is unknown. Given the general function of PABPN1 in RNA metabolism, intrinsic characteristics of skeletal muscle may make this tissue susceptible to the effects of mutant PABPN1.

**Methods:**

To begin to understand the muscle specificity of OPMD, we investigated the steady-state levels of PABPN1 in different tissues of humans and mice. Additionally, we analyzed the levels of PABPN1 during muscle regeneration after injury in mice. Furthermore, we assessed the dynamics of PABPN1 mRNA decay in skeletal muscle compared to kidney.

**Results:**

Here, we show that the steady-state levels of both PABPN1 mRNA and protein are drastically lower in mouse and human skeletal muscle, particularly those impacted in OPMD, compared to other tissues. In contrast, PABPN1 levels are increased during muscle regeneration, suggesting a greater requirement for PABPN1 function during tissue repair. Further analysis indicates that modulation of PABPN1 expression is likely due to post-transcriptional mechanisms acting at the level of mRNA stability.

**Conclusions:**

Our results demonstrate that PABPN1 steady-state levels and likely control of expression differ significantly in skeletal muscle as compared to other tissues, which could have important implications for understanding the muscle-specific nature of OPMD.

## Background

RNA-binding proteins regulate all steps of RNA biogenesis and play a key role in post-transcriptional regulation of gene expression [1]. Key players among these RNA-binding proteins are the poly(A)-binding proteins, which modulate 3′-end formation of mRNA transcripts [2]. The nuclear poly (A)-binding protein 1 (PABPN1) is a ubiquitously expressed protein in eukaryotes that binds with high affinity to polyadenosine RNA [2]. PABPN1 has critical roles at multiple steps in post-transcriptional regulation of gene expression. The best characterized role of PABPN1 is in mRNA polyadenylation, where PABPN1 stimulates poly(A) synthesis by direct interaction with the nascent mRNA poly(A) tail and the poly(A) polymerase [3,4]. In a subsequent regulatory step, PABPN1 decreases poly(A) polymerase elongation activity following addition of 250 adenine residues, thereby dictating the length of the poly(A) tail added to mRNA transcripts [4]. Moreover, PABPN1 is also involved in regulation of alternative cleavage and polyadenylation by suppressing weak proximal polyadenylation signals [5], which can influence both gene expression and the structure of the protein produced [6]. Finally, PABPN1 has been implicated in the polyadenylation-dependent pathway of RNA decay, which targets non-protein coding genes such as the long non-coding RNAs (lncRNAs) [7]. Thus, PABPN1 modulates a number of processes that are critical for controlling gene expression.

In addition to playing a key role in RNA metabolism, PABPN1 is of significant clinical interest as mutations in the *PABPN1* gene lead to oculopharyngeal muscular dystrophy (OPMD) [8]. This disease is caused by a small GCN trinucleotide expansion in the coding region of *PABPN1*, resulting in the expansion of a stretch of 10 alanines to 12 to 17 alanines in the PABPN1 N-terminus. OPMD is a late onset, autosomal dominant disease characterized primarily by progressive eyelid drooping (ptosis) and difficulties in swallowing [9]. Additional weakness is noted in proximal limb, facial and other extraocular muscles [10-12]. Disease progression is variable between patients and complications include choking, regurgitation, aspiration and pneumonia. The pathologic hallmark of the disease is the presence of nuclear aggregates of PABPN1 in muscle [13,14]. Given the ubiquitous expression and general function of PABPN1 in RNA metabolism [15], how mutations of this post-transcriptional regulatory factor cause a muscle-specific disease is unclear. PABPN1 is essential for both mRNA biogenesis as well as proliferation and differentiation of myogenic precursor cells, suggesting a critical role in muscle regeneration and maintenance [16]. Skeletal muscle is highly specialized for contraction and has unique characteristics compared to other tissues, such as being highly regenerative and comprised of multinucleated post-mitotic cells, which suggests that intrinsic characteristics of this tissue may make it more vulnerable to the effects of mutant PABPN1 than other tissues that are not affected in OPMD.

To begin to understand the muscle specificity of OPMD, we investigated the steady-state levels of the PABPN1 protein in different tissues. We find that the steady-state levels of PABPN1 are drastically lower in skeletal muscle compared to other tissues. Strikingly, craniofacial muscles, which are affected in OPMD, show the lowest levels of PABPN1. We also found that PABPN1 is upregulated during muscle repair after injury. Studies of mRNA stability indicate that regulation of PABPN1 expression is likely due to distinct post-transcriptional mechanisms in different tissues. Taken together, our results demonstrate that PABPN1 steady-state levels and likely control of expression differ significantly in skeletal muscle as compared to other tissues, which could have important implications for understanding the muscle-specific nature of OPMD.

## Methods

### Animals and primary muscle cell culture

Experiments involving animals were performed in accordance with approved guidelines and ethical approval from Emory University’s Institutional Animal Care and Use Committee. Adult male C57BL/6 mice between 2 to 6 months of age were used in experiments. To induce regeneration, gastrocnemius muscles of male mice were injected with 40 μl of 1.2% BaCl_2_[17] and collected 2, 5 and 14 days after injury. Primary myoblasts were derived from the hindlimb muscles of mice and cultured to >99% purity as previously described [18]. Cells were maintained in growth media (GM: Ham’s F10, 20% fetal bovine serum, 5 ng/ml basic fibroblast growth factor (bFGF), 100 U/ml penicillin G, 100 mg/ml streptomycin) in a humidified 5% CO_2_ incubator at 37°C on collagen-coated dishes. For histologic analyses, serial 10 μm sections were collected along the length of the muscle and stained with hematoxylin and eosin. Images were obtained using Axiovert 200 M microscope with a 0.30 NA 10× or 20× Plan-Neofluar objective (Carl Zeiss MicroImaging, Inc., Oberkochen, Germany) and camera (QImaging, Surrey, Canada) with OpenLab 5.5.2 (PerkinElmer, Waltham, MA).

### Immunoblot analysis

Tissues were homogenized in radioimmunoprecipitation assay (RIPA)-2 buffer (50 mM Tris-HCl pH 8.0, 150 mM NaCl, 1% NP-40, 0.5% deoxycholic acid, 0.1% SDS) with protease inhibitors (Complete Mini Tablets, Roche, Pleasanton, CA). Equal amounts of total protein were resolved by SDS-PAGE, transferred to nitrocellulose and the desired protein was detected by immunoblotting with appropriate antibodies and enhanced chemiluminescence. PABPN1 levels in human tissues were analyzed using INSTA-blot (IMGENEX, San Diego, CA) membranes. The primary antibodies and concentrations used were as follows: anti-PABPN1 antibody 1:5,000 [16], anti-Histone H3 1:1,000 (Abcam, Cambridge, MA), anti-heat shock protein 90 (HSP90) 1:1,000 (Santa Cruz Biotechnology, Dallas, TX) and anti-tubulin 1:5,000 (Sigma-Aldrich, St. Louis, MO). Anti-mouse or anti-rabbit IgG 1:5,000 (Jackson ImmunoResearch, West Grove, PA) were used as secondary antibodies.

### Northern blot analysis

Total RNA from tissues, primary cell cultures and fluorescence-activated cell sorting (FACS)-sorted cells was isolated using TRIzol (Invitrogen, Carlsbad, CA) according to the manufacturer’s protocol. Northern blotting was performed as described previously by Ausubel *et al*. [19]. DNA probes were generated by polymerase chain reaction (PCR) using custom primers (PABPN1-F 5′-CCCAGGCAATGCTGGCCCAGTGATCATGTCTC-3′ and PABPN1-R 5′-CTAGCCCGGCCCCTGTAGATTCGACCCCGGGGC-3′, c-Myc-F 5′-GAACTTCACCAACAGGAACTATGACCTCG-3′ and c-Myc-R 5′-GGTGTCTCCTCATGCAGCACTAGG-3′), primers from SA Biosciences, Valencia, CA (peroxisome proliferator-activated receptor gamma coactivator 1α (PGC1α); PPM03360E, glyceraldehyde 3-phosphate dehydrogenase (GAPDH); PPM02946E) or by Ambion, Austin, TX (QuantumRNA Classic II 18S). PCR products were labeled with [α-^32^P]dCTP using a random primer DNA labeling system (Invitrogen, Carlsbad, CA).

### FACS

Mononucleated cells were enzymatically isolated from gastrocnemius muscles 3 days after BaCl_2_ injury and fluorescently labeled with antibodies to CD31 and CD45 (PE), Sca-1 (PE-Cy7), and alpha-7-integrin (AlexaFluor 649). Propidium iodide staining was used to gate out dead cells from the sort. Myoblasts (CD31^**-**^/CD45^**-**^/Sca-1^**-**^/alpha-7-integrin^**+**^/PI^**-**^) and non-myogenic cells (CD31^**+**^/CD45^**+**^/Sca-1^**+**^/alpha-7-integrin^**-**^/PI^**-**^) were collected using a FACSAria II (Becton-Dickinson, Franklin Lakes, NJ). Isolated cells were then processed for RNA extraction.

### Quantitative reverse transcription (RT)-PCR

cDNA synthesis from 100 ng RNA was performed using M-MLV reverse transcriptase (Invitrogen, Carlsbad, CA). mRNA levels were determined by real-time PCR using the iQ SYBR Green (Bio-Rad, Hercules, CA) and iCycler iQ Real-Time Detection System and software (Bio-Rad, Hercules, CA). The relative levels of PABPN1 were determined by the ΔΔCt method and normalized to the housekeeping gene HPRT1. Primers were from SA Bioscences, Valencia, CA (PABPN1: PPM25445A, HPRT1: PPM03559E).

### mRNA decay

To analyze mRNA stability *in vivo*, mice were injected intraperitoneally with actinomycin D (Sigma-Aldrich, St. Louis, MO) at 2.5 μg/g and quadriceps muscles and kidney were collected 1, 2, 4 and 6 h later. To measure mRNA stability in primary myoblasts *in vitro*, 5 μg/ml actinomycin D was added to the growth medium and cells were harvested 0.5, 1, 2 and 4 h later. Total RNA was extracted from tissues or cells and analyzed by northern blot and half-lives were determined by densitometry.

### 5′ and 3′ RACE

In order to determine the 5′ and 3′UTRs of PABPN1 transcripts, we used the 5′ and 3′ rapid amplification of cDNA ends (RACE) system (Invitrogen, Carlsbad, CA), respectively. Total RNA from either muscle or testis was used as a template according to the manufacturer’s instructions. PCR products were cloned into the pCR2.1 vector (TOPO TA cloning, Invitrogen, Carlsbad, CA) and sequenced by Beckman Coulter Genomics, Danvers, MA.

### Statistical analysis

Statistical analysis to determine significance between two groups was performed using a Student’s t test. One-way analysis of variance (ANOVA) was used for comparisons between multiple groups as appropriate. All statistical analyses were performed using GraphPad Prism 5.0 for Macintosh (GraphPad Software). Differences were considered to be statistically significant at *P* <0.05.

## Results

### PABPN1 levels are lower in skeletal muscle compared to other tissues

**Figure 1:**
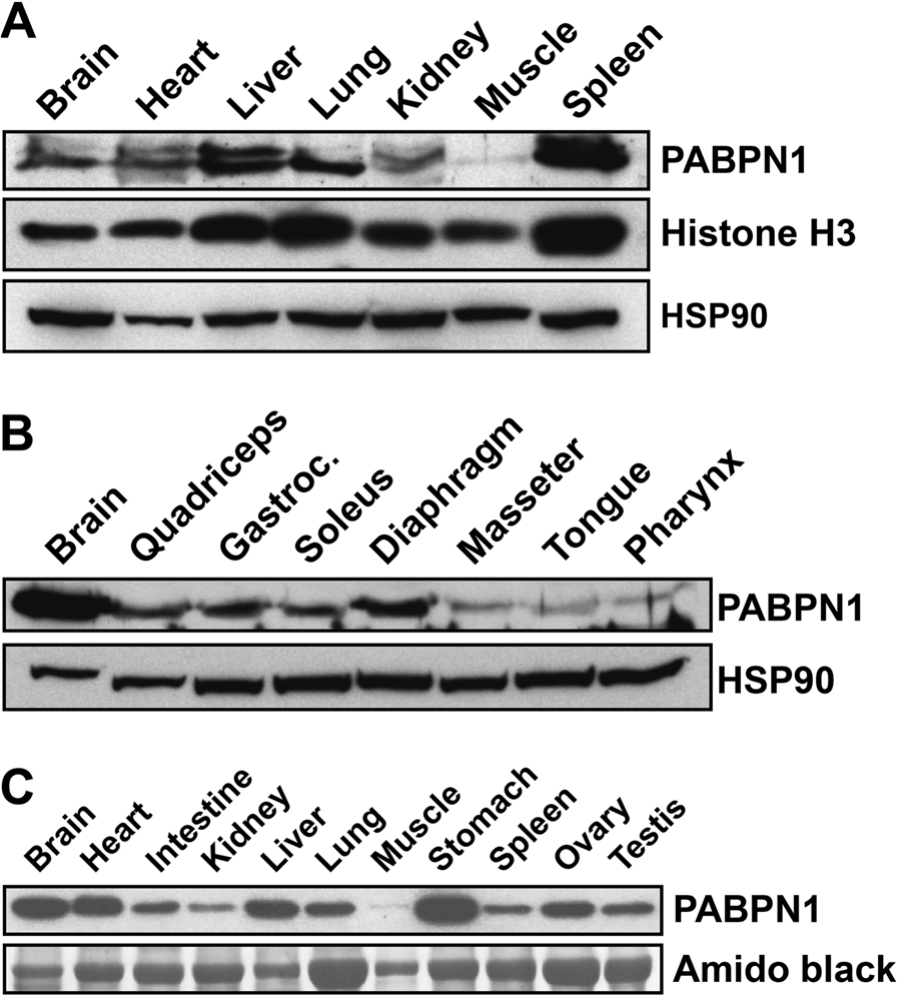
**Nuclear poly(A) binding protein 1 (PABPN1) levels are low in all skeletal muscles.** Lysates prepared from different **(A)** mouse tissues (50 μg of total protein per lane), **(B)** mouse muscles (150 μg of total protein per lane) or **(C)** human tissues (20 μg of total protein per lane) were immunoblotted with anti-PABPN1 antibody. Histone H3 and heat shock protein 90 (HSP90) were used as loading controls for mouse samples. Amido black staining was used as the loading control for human samples. Immunoblots are representative of at least three independent sets of tissues.

A better understanding of the mechanisms that underlie OPMD pathology can be obtained by analyzing the function of PABPN1 in skeletal muscle. To begin to identify muscle-specific properties of PABPN1, we first examined the expression of PABPN1 across different tissues. Immunoblot analysis revealed that PABPN1 steady-state levels vary significantly among mouse tissues, with skeletal muscle displaying the lowest levels of PABPN1 (Figure  1A). The low abundance of PABPN1 in skeletal muscle could result from skewed misrepresentation of this protein within the protein pool by the uniquely high levels of cytoplasmic proteins comprising the contractile machinery in this tissue. However, relatively similar levels of both the nuclear protein histone H3 [20] and the cytoplasmic protein HSP90 [21] were observed between muscle and other tissues, suggesting that the nuclear protein fraction is not under-represented in muscle. Furthermore, analysis of PABPN1 levels among different mouse muscles revealed even lower levels of this protein in the craniofacial muscles (masseter, tongue and pharynx), some of which are muscles primarily affected in OPMD patients [12], compared to other muscles of the body (Figure  1B). Significantly lower levels of PABPN1 in muscle as compared to other tissues were also observed in human samples (Figure  1C), suggesting that the low levels of this protein in muscle are not species-specific findings, and this may have physiologic implications for humans.

**Figure 2:**
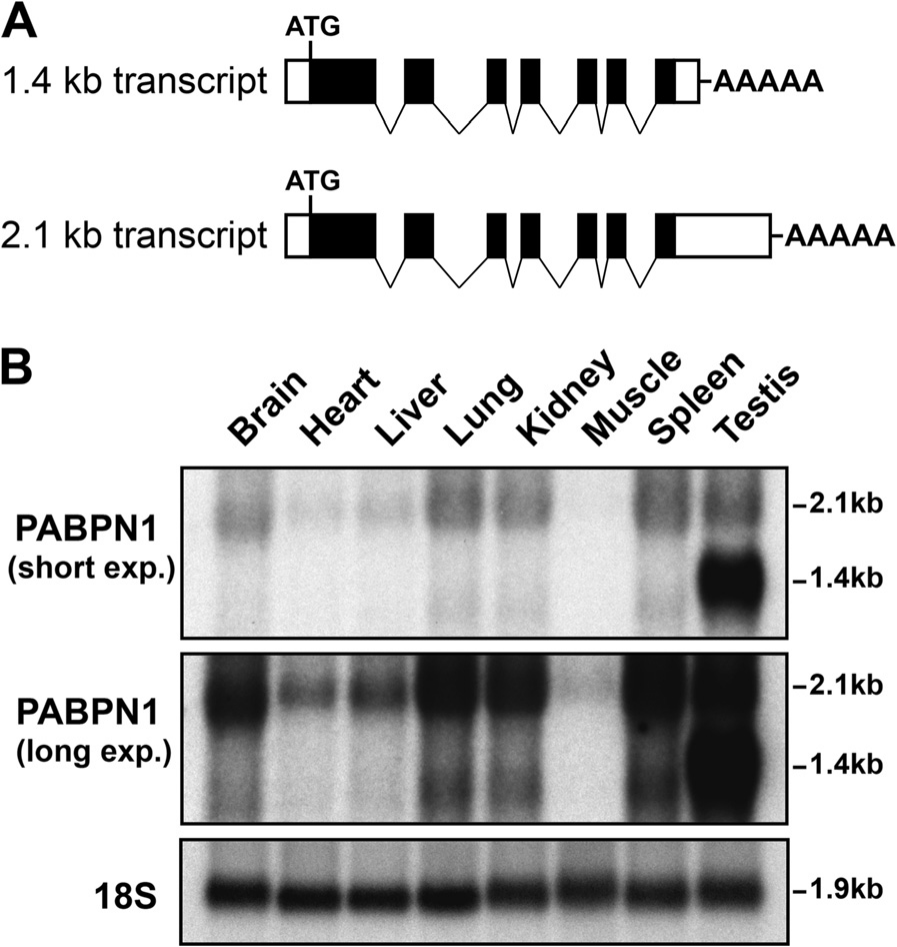
**Nuclear poly(A) binding protein 1 (PABPN1) mRNA levels are low in skeletal muscle. (A)** Structure of the 2.1 kb and 1.4 kb PABPN1 transcripts (solid boxes represent coding regions and open boxes non-coding regions). **(B)** Total RNA from different mouse tissues was analyzed by northern blot using a PABPN1 probe. Two different exposures, short and long, are shown. 18S rRNA was probed as a loading control. Figure is representative of at least three independent sets of tissues.

To examine whether the expression of PABPN1 is regulated at the protein or RNA level we performed northern blot analysis (Figure  2). This analysis revealed a strong correlation between the low levels of PABPN1 protein and the low abundance of PABPN1 transcript in mouse skeletal muscle (Figure  2B), suggesting that control of PABPN1 expression occurs at the RNA level, either by transcriptional or post-transcriptional means. As previously reported, PABPN1 has two major mRNA variants, a 2.1 kb and a 1.4 kb transcript (Figure  2) [22,23]. The 2.1 kb transcript, which was detected in all tissues but was present at low levels in muscle (Figure  2B), utilizes a distal polyadenylation site 851 bp downstream of the stop codon (Figure  2A) [23]. The 1.4 kb represents the transcript that uses a proximal polyadenylation site 66 bp downstream of the stop codon (Figure  2A) [23]. This 1.4 kb mRNA variant was the predominant transcript only in testis, but was also found in other tissues at much smaller amounts (Figure  2B). Interestingly, the levels of the 1.4 kb PABPN1 transcript were very high in testis, which correlates with the very high levels of PABPN1 protein observed in this tissue (Additional file 1: Figure S1). Furthermore, with the exception of testis, no significant variation in the ratio between the two mRNA isoforms was observed in the analyzed tissues. Northern blotting also revealed a band of approximately 3.6 kb that was observed in all tissues (data not shown). This transcript was previously reported and suggested to be either a transcript of a related gene [22] or generated by a distinct *PABPN1* promoter [23]. We performed both 5′RACE and 3′RACE from kidney, muscle and testis but failed to identify any novel PABPN1 transcript other than the 1.4 kb and 2.1 kb mRNAs variants, suggesting the 3.6 kb band might indeed represent a transcript from a related gene. Together, our results from immunoblotting and northern blotting reveal low steady-state levels of PABPN1 mRNA and protein in skeletal muscle, which is indicative of either a decrease in PABPN1 transcription or altered mRNA stability in this tissue.

### PABPN1 levels are increased during muscle regeneration

**Figure 3:**
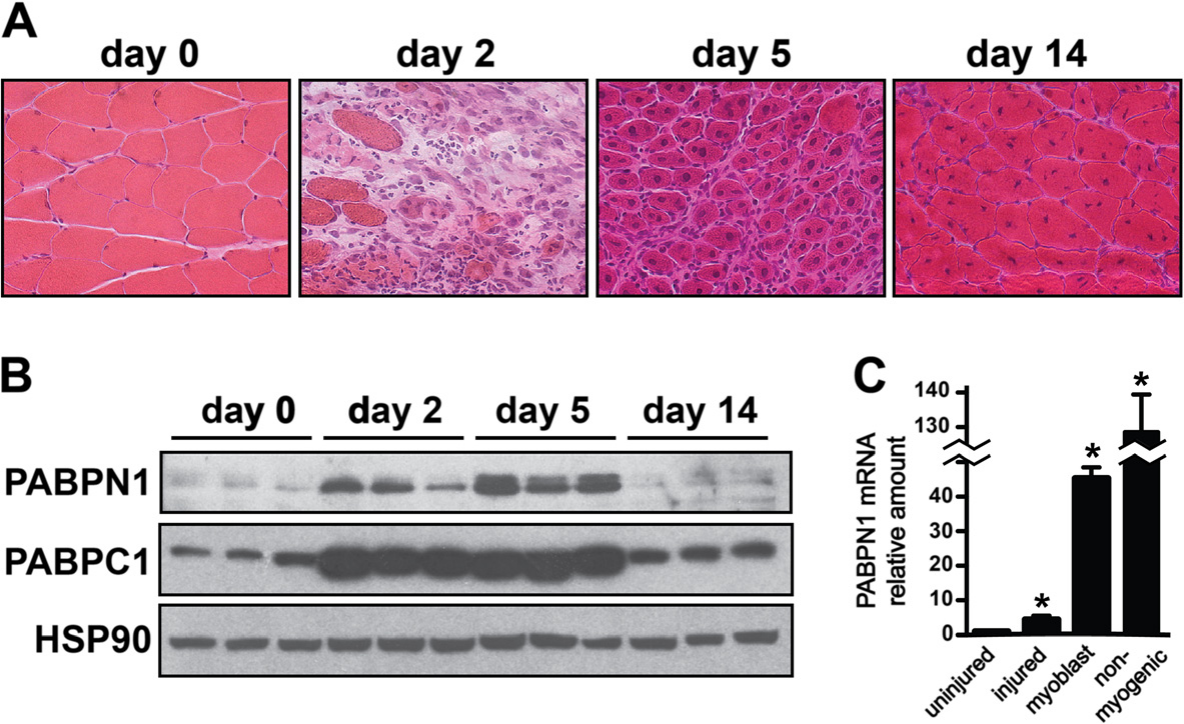
**Nuclear poly(A) binding protein 1 (PABPN1) levels are increased during muscle regeneration in part due to increased levels in myoblasts. (A)** Representative hematoxylin and eosin stained sections of gastrocnemius muscles at different times after BaCl_2_ injury are shown. **(B)** Lysates were prepared from gastrocnemius muscles at different times after injury and were immunoblotted with anti-PABPN1, PABPC1 or heat shock protein 90 (HSP90) antibodies (n = 3 per timepoint). **(C)** Total RNA was obtained from uninjured and injured muscle tissue (three independent samples) as well as fluorescence-activated cell sorting (FACS)-sorted myoblasts and non-myogenic cells obtained 3 days after muscle injury (pooled from five mice). PABPN1 mRNA levels were determined using real-time polymerase chain reaction (PCR) and hypoxanthine-guanine phosphoribosyltransferase (HPRT) mRNA was used as an internal control. Amount of PABPN1 mRNA relative to uninjured muscle is shown; n = 3. Data are mean ± SD; *P <0.05 vs uninjured muscle.

Adult skeletal muscle is comprised primarily of post-mitotic myofibers, however, it is a highly regenerative tissue that undergoes extensive repair after injury (Figure  3A) [24]. In the earliest phases of muscle regeneration, inflammatory cells invade the tissue to remove dead tissue. Subsequently, large numbers of proliferative myoblasts derived from resident stem cells undergo differentiation and fusion to form new myofibers. Although PABPN1 levels are very low in adult muscle tissue, levels were significantly increased during the period of extensive cellular proliferation, differentiation and fusion that occurs 2 to 5 days after muscle injury (Figure 3B). However, 14 days after injury, when muscle architecture was restored (Figure  3A), PABPN1 levels were again low (Figure  3B). A similar pattern of upregulation was observed for the cytoplasmic poly(A) binding protein, PABPC1 [2], during muscle regeneration, however the levels of the heat shock protein HSP90 remained constant over the time course. This result indicates that increased levels of poly(A)-binding proteins during muscle regeneration are not unique to PABPN1. To determine whether the increased levels of PABPN1 observed at 2 to 5 days after injury were due in part to myoblasts, we used flow cytometry and specific antibodies to isolate myoblasts and non-myogenic cells (including inflammatory cells) from mouse muscles 3 days after injury. As we were unable to perform immunoblots for PABPN1 on the small amount of cells isolated by flow cytometry, we used quantitative RT-PCR to examine PABPN1 transcript levels in sorted cells compared to muscle tissue. Similar to what we observed for PABPN1 protein, PABPN1 transcript levels were increased approximately fivefold in injured compared to uninjured muscle (Figure  3C). We also found that PABPN1 mRNA levels were exceptionally high in both sorted myoblasts (CD31^**-**^/CD45^**-**^/Sca-1^**-**^/alpha-7-integrin^**+**^) and non-myogenic cells (CD31^**+**^/CD45^**+**^/Sca-1^**+**^/alpha-7-integrin^**-**^) compared to uninjured muscle tissue (Figure  3C). These data suggest that myoblasts significantly contribute to increased PABPN1 levels in regenerating muscle. We conclude that PABPN1 levels are not static in muscle but rather modulated by the physiologic state of the tissue, suggesting a greater requirement for PABPN1 function during tissue repair.

### PABPN1 mRNA is unstable in skeletal muscle

**Figure 4:**
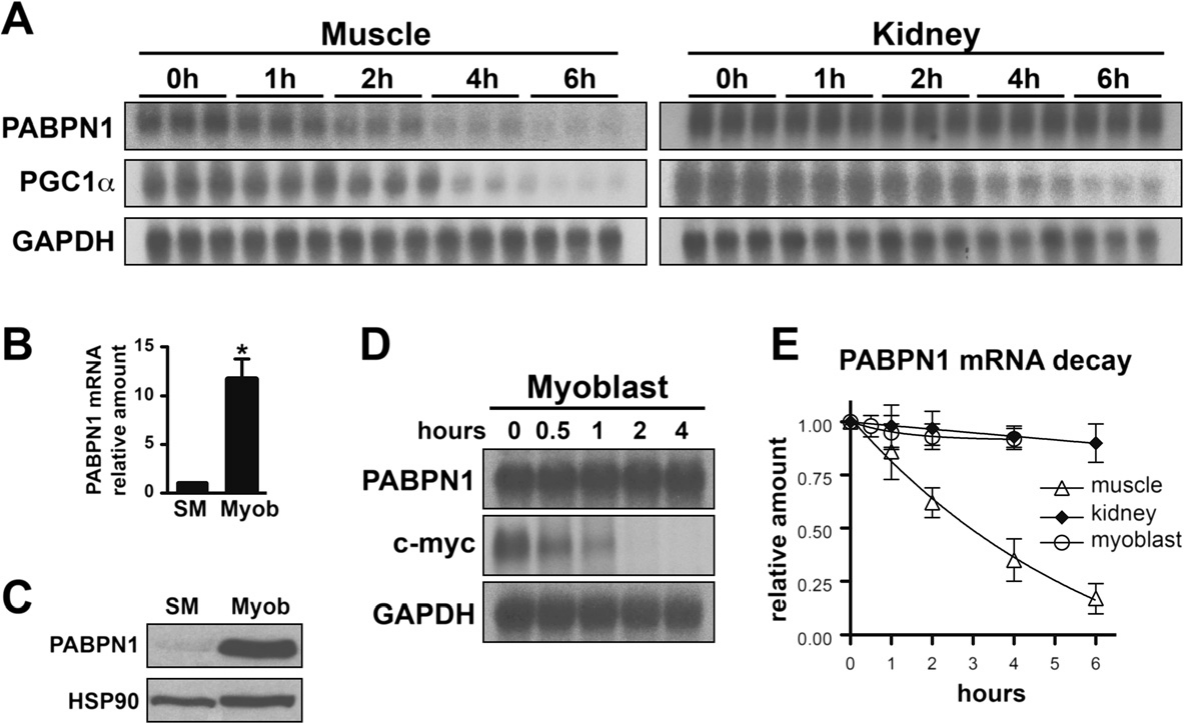
**Nuclear poly(A) binding protein 1 (PABPN1) mRNA is unstable in muscle tissue but stable in cultured myoblasts. (A)** Total RNA was collected at different timepoints after injection of actinomycin D to inhibit transcription and PABPN1 mRNA decay was analyzed by northern blot. Time courses are shown for samples from muscle and kidney. Peroxisome proliferator-activated receptor gamma coactivator 1α (PGC1α) and glyceraldehyde 3-phosphate dehydrogenase (GAPDH), a known unstable and stable transcript, respectively, were probed as controls (n = 3 per timepoint). To visualize PABPN1 signal in muscle samples, the blot was exposed significantly longer than for kidney samples. **(B)** Total RNA was obtained from skeletal muscle (SM) and cultured primary mouse myoblasts (Mb) and PABPN1 mRNA levels were determined using real-time polymerase chain reaction (PCR) and hypoxanthine-guanine phosphoribosyltransferase (HPRT) mRNA was used as an internal control. The amount of PABPN1 mRNA relative to skeletal muscle (SM) is shown; n = 3 independent samples. Data are mean ± SD; *P <0.05 vs skeletal muscle. **(C)** Protein extracts were prepared from SM and Mb and immunoblotted with anti-PABPN1 antibody. HSP90 was used as a loading control. The immunoblot is representative of at least three independent samples. **(D)** Total RNA was collected at different timepoints after treatment of cultured primary mouse myoblasts with actinomycin D and PABPN1 mRNA decay was analyzed by northern blot; c-Myc and GAPDH, known unstable and stable transcripts in myoblasts, respectively, were probed as controls. Averages of densitometric measurements of northern blot bands were used to determine mRNA decay. The image is representative of at least three independent samples. **(E)** The decay profile of PABPN1 mRNA in muscle, kidney and cultured myoblasts plotted as mRNA amount relative to timepoint T = 0 h (n = 3 samples per timepoint). Data are mean ± SD.

The two PABPN1 transcripts schematized in Figure  2A arise from the usage of two different polyadenylation sites. The smaller 1.4 kb transcript contains virtually no 3′UTR, whereas the longer 2.1 kb transcript harbors a 3′UTR of 851 bp containing a putative mRNA AU-rich destabilizing element (ARE) [23]. We hypothesized that the 2.1 kb transcript, which is the most abundant transcript in all tissues but testis and contains a putative ARE, may be subject to post-transcriptional regulation in different tissues. To assess if this transcript is differently regulated in muscle, we analyzed PABPN1 mRNA stability in muscle and kidney after blocking transcription in mice with actinomycin D. We observed that the half-life of the 2.1 kb PABPN1 transcript was significantly shorter in muscle (2.3 h) compared to kidney (>6 h) (Figure  4A,E; Table  1). As a control to demonstrate similar transcriptional inhibition between both tissues, we analyzed the stability of PGC1α and GAPDH mRNAs, known unstable and stable transcripts, respectively [25,26]. As expected, PGC1α mRNA displayed a short half-life in both muscle and kidney (1.7 h and 2.6 h) compared to GAPDH mRNA (>>6 h in both tissues) (Figure  4A,E; Table  1). These results demonstrate a strong correlation between the high steady-state levels of PABPN1 protein and the stable transcript in kidney, whereas in skeletal muscle, the low steady-state levels of PABPN1 correlate with the unstable PABPN1 transcript.

**Table 1:** Nuclear poly(A) binding protein 1 (PABPN1) mRNA is unstable in muscle tissue but stable in cultured myoblasts

	**Muscle**	**Kidney**	**Myoblast**
**PABPN1**	2.3 h	>>6 h	>>6 h
**PGC1α**	1.7 h	2.6 h	ND
**GAPDH**	>>6 h	>>6 h	>>6 h
**c-Myc**	ND	ND	0.3 h

*GAPDH* glyceraldehyde 3-phosphate dehydrogenase, *ND* not determined, *PGC1α* peroxisome proliferator-activated receptor gamma coactivator 1α.

As shown earlier (Figure  3C), PABPN1 levels are modulated during muscle regeneration and myoblasts contribute in part to the increased levels of PABPN1 during this process. We next investigated if the increase in PABPN1 levels in myoblasts is accompanied by a corresponding increase in PABPN1 mRNA stability. Similar to myoblasts directly isolated from injured muscle, we found that cultured primary mouse myoblasts displayed higher levels of PABPN1 mRNA compared to uninjured muscle tissue (Figure  4B). Consistent with this finding, steady-state levels of PABPN1 protein were also higher in cultured myoblasts compared to muscle tissue (Figure  4C). We analyzed the stability of PABPN1 transcripts in cultured myoblasts. As observed in skeletal muscle tissue, the 2.1 kb PABPN1 transcript was the predominant transcript in myoblasts (data not shown). However, in contrast to muscle tissue, the 2.1 kb PABPN1 transcript was extremely stable in myoblasts (Figure  4D,E; Table 1). As expected, c-Myc mRNA, a known unstable transcript in myoblasts [27], had a short half-life compared to the much longer half-life for GAPDH mRNA, a known stable transcript (Table  1). These results indicate that the high levels of PABPN1 in cultured myoblasts, and likely in myoblasts present during muscle regeneration, is due at least in part to the increase in PABPN1 mRNA stability in those cells compared to muscle tissue. Taken together, our results suggest that PABPN1 expression in different tissues or during muscle regeneration is regulated by a post-transcriptional mechanism that modulates transcript stability.

## Discussion

Studying PABPN1 specifically in skeletal muscle is critical for defining the mechanisms which make this tissue uniquely susceptible to the mutation causing OPMD. Here, we report that steady-state levels of PABPN1 mRNA and protein are low in skeletal muscle and that expression of PABPN1 in this tissue is controlled, at least in part, by post-transcriptional regulation of RNA levels. We also demonstrate that PABPN1 levels are modulated during muscle repair providing further support for regulation of PABPN1 expression in this tissue.

PABPN1 is not the only ubiquitous protein with a general function in basic cellular processes whose expression level is variable among tissues [28,29]. For example, the expression of histone H3A, transcription elongation factor A1 (TCEA1) and heterogeneous nuclear ribonucleoprotein (hnRNP) C is relatively constant among different tissues, whereas levels of GAPDH, β-actin and histone H2A are among the most variable within tissues [29]. These differences in expression levels are most likely related to intrinsic properties of individual tissues and reflect differences in metabolic activity and cellular structure.

The extremely low levels of PABPN1 in skeletal muscle compared to other tissues may indicate a low requirement for this factor in basal muscle metabolism and maintenance. Skeletal muscle is distinctly characterized by multinucleated, post-mitotic cells with a very specialized function and low complexity transcriptome [30,31]. In skeletal muscle, a small number of genes contribute to a large fraction of the total mRNA pool, with the ten most expressed genes in muscle accounting for 20% to 40% of the total mRNA [31]. The most abundant transcripts in skeletal muscle encode proteins involved in contraction, glucose metabolism, ATP production and ribosomal proteins [30,31], consistent with the role of this tissue in movement and metabolism. Such transcripts encoding proteins involved in general cellular functions are usually stable with low turnover [27,32]. Therefore, the low complexity of the skeletal muscle transcriptome associated with low turnover of a significant fraction of its transcripts may explain why skeletal muscle has low requirements for a protein involved in mRNA metabolism such as PABPN1.

Our data indicate the low levels of PABPN1 in skeletal muscle are, at least in part, determined at the level of regulation of PABPN1 transcript stability. Regulation of mRNA decay rate is a key factor in determining the expression pattern of many genes allowing rapid adaptation to changing cellular requirements [33,34]. PABPN1 levels increase significantly during skeletal muscle regeneration suggesting a greater requirement for PABPN1 in myoblasts and non-myogenic cells such as inflammatory cells, which may be due to their highly proliferative status and to a more complex transcriptome compared to uninjured muscle tissue. As the increased levels of PABPN1 in regenerating muscle correlate with an increased transcript stability in myoblasts and subsequent increase in the steady-state levels of PABPN1 transcript, we suggest that skeletal muscle employs a post-transcriptional mechanism to control PABPN1 levels according to the tissue requirements.

mRNA decay rates are modulated by an interplay of specific stabilizing or destabilizing factors with the transcript, such as RNA-binding proteins and/or miRNAs and their associated enzymes [35]. One of the most studied post-transcriptional pathways is orchestrated by a variety of RNA-binding proteins that interact with AU-rich elements (ARE) within the 3′UTR of mRNAs [33,36] and many unstable mRNAs expressed in muscle contain AU-rich elements in their 3′UTRs [27]. As the main 2.1 kb PABPN1 transcript expressed in skeletal muscle harbors an ARE in the 3′UTR, we speculate this pathway is a strong candidate for the control of PABPN1 levels in skeletal muscle.

**Figure 5:**
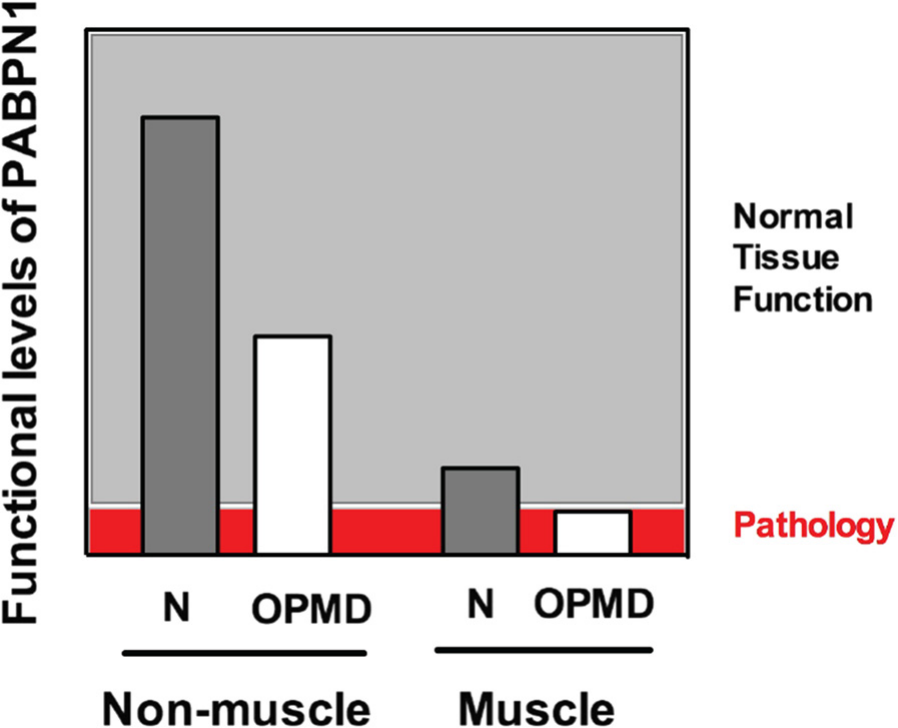
**Low levels of nuclear poly(A) binding protein 1 (PABPN1) in skeletal muscle may predispose this tissue to the deleterious effects of alanine-expanded PABPN1.** We show muscle has lower levels of PABPN1 compared to other tissues in normal individuals (N) but these levels are adequate for normal tissue function. In patients with oculopharyngeal muscular dystrophy (OPMD), functional levels of PABPN1 could be decreased in all tissues due to expression of mutant PABPN1. However, muscle-specific pathology ensues in autosomal dominant OPMD because the levels of PABPN1 fall below the threshold required to maintain proper tissue function.

The specific PABPN1 expression pattern observed in skeletal muscle may be an important feature that makes this tissue more susceptible than others to the mutations in PABPN1 that cause the muscle-specific disease OPMD. Whether the alanine expansion in PABPN1 leads to a gain-of function or loss-of-function of this protein is unknown [12,15]. The nuclear aggregates observed in muscle of OPMD patients may exert toxic effects in the tissue as hypothesized for other polyglutamine and polyalanine expansion disorders [37-40]. However, as wild-type PABPN1 can form reversible aggregates in neurons in response to changes in cell physiology without overt pathology [41], the toxicity of PABPN1 nuclear aggregates is unlikely to be the exclusive cause of OPMD etiology. In the loss-of-function model of OPMD etiology, one mechanism that could lead to a loss or decrease of PABPN1 function is an intrinsic reduction in PABPN1 activity caused by the alanine expansion. Although wild-type and mutant PABPN1 appear to have similar polyadenylation activity *in vitro*[2], the effects of the alanine expansion on this or other PABPN1 functions have not yet been addressed in the context of skeletal muscle *in vivo*. Another mechanism that could explain a loss of PABPN1 function is the depletion of the soluble and functional fraction of PABPN1 by sequestration in the nuclear aggregates present in muscle of OPMD patients. In fact, a recent study that examined PABPN1 transcript levels in human muscle samples reported a decrease in steady-state levels of PABPN1 mRNA after the fifth decade of life, the common age for onset of OPMD symptoms [42]. This study also presented evidence that this decrease in PABPN1 transcript levels is accelerated in OPMD patients [42]. These recent findings support the idea that a loss of PABPN1 function could contribute to the muscle-specific pathology in OPMD. Consistent with this idea, overexpression of wild-type PABPN1 reduces the pathology caused by the expression of alanine-expanded PABPN1 in both cell and mouse models of OPMD [43]. In these loss-of-function scenarios, the low level of PABPN1 in the skeletal muscle may make this tissue specifically more vulnerable to a further decrease in the total amount of functional PABPN1 available as illustrated in the schematic in Figure  5. The resulting lowered levels of PABPN1 may then be below the threshold amount of PABPN1 activity that is required for proper tissue maintenance leading to pathology.

## Conclusions

Our results demonstrate that PABPN1 steady-state levels and likely control of expression differ significantly in skeletal muscle as compared to other tissues, which could have important implications for understanding the muscle-specific nature of OPMD. We suggest the low levels of PABPN1 observed in skeletal muscle may be an important aspect of this tissue that underlies OPMD pathology. Further studies are necessary to better comprehend the mechanisms and implications of the regulation of PABPN1 expression in skeletal muscle, which could open avenues for potential therapeutic approaches for OPMD.

## Abbreviations

ARE: AU-rich element; GAPDH: glyceraldehyde-3-phosphate dehydrogenase; HSP90: Heat shock protein 90; OPMD: Oculopharyngeal muscular dystrophy; PABPN1: Nuclear poly(A)-binding protein 1; PGC1α: Peroxisome proliferator-activated receptor gamma, coactivator 1α; qRT PCR: Quantitative real-time polymerase chain reaction; RACE: Rapid amplification of cDNA ends; UTR: Untranslated region.

## Competing interests

The authors declare that they have no competing interests.

## Authors' contributions

LHA, AHC and GKP conceived and designed the study. LHA performed the research. LHA, AHC and GKP analyzed the research and wrote the manuscript. All authors read and approved the final manuscript.

## Supplementary Material

Additional file 1: Figure S1 Nuclear poly(A) binding protein 1 (PABPN1) levels are high in testis. Lysates prepared from mouse kidney, testis and skeletal muscle were immunoblotted with anti-PABPN1 antibody, and heat shock protein 90 (HSP90) was used as loading controls. Immunoblots are representative of at least three independent samples. Click here for file
