# Supplementary figures and images for: Control of mRNA stability contributes to low levels of nuclear poly(A) binding protein 1 (PABPN1) in skeletal muscle
Source: Skelet Muscle. 2013 Oct 1;3:23. doi: 10.1186/2044-5040-3-23 (PMC3879409; doi:10.1186/2044-5040-3-23)

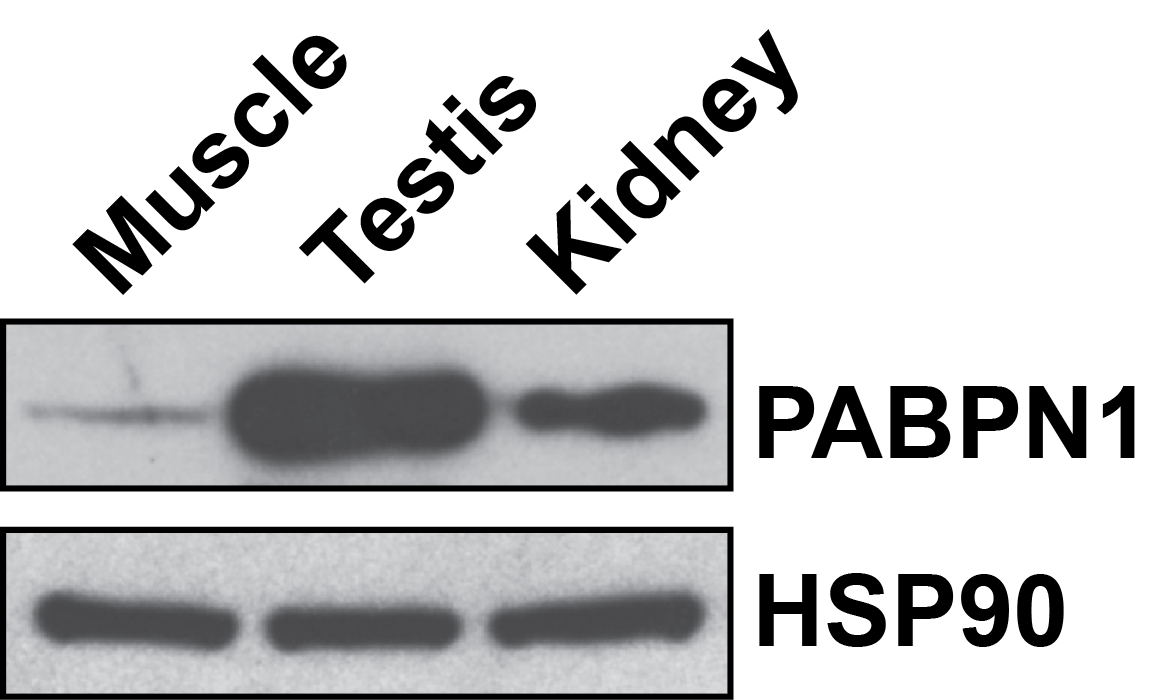

Supplement: Additional file 1: Figure S1 — Nuclear poly(A) binding protein 1 (PABPN1) levels are high in testis. Lysates prepared from mouse kidney, testis and skeletal muscle were immunoblotted with anti-PABPN1 antibody, and heat shock protein 90 (HSP90) was used as loading controls. Immunoblots are representative of at least three independent samples. [file 2044-5040-3-23-S1.png]
